# Supplementary material for: The WNK-OXSR1 osmosensing pathway mediates intestinal regeneration via Hippo-YAP signaling
Source: EMBO J. 2026 Mar 18;45(8):2456–93. doi: 10.1038/s44318-026-00738-8 (PMC13083915; doi:10.1038/s44318-026-00738-8)
Supplement: Supplementary file 1 — Appendix [file 44318_2026_738_MOESM1_ESM.pdf]

Appendix for

The WNK-OXSR1 osmosensing pathway mediates intestinal regeneration via Hippo-YAP Signaling

Contents

Appendix Figure S1 2

Appendix Figure S2 4

Appendix Figure S1

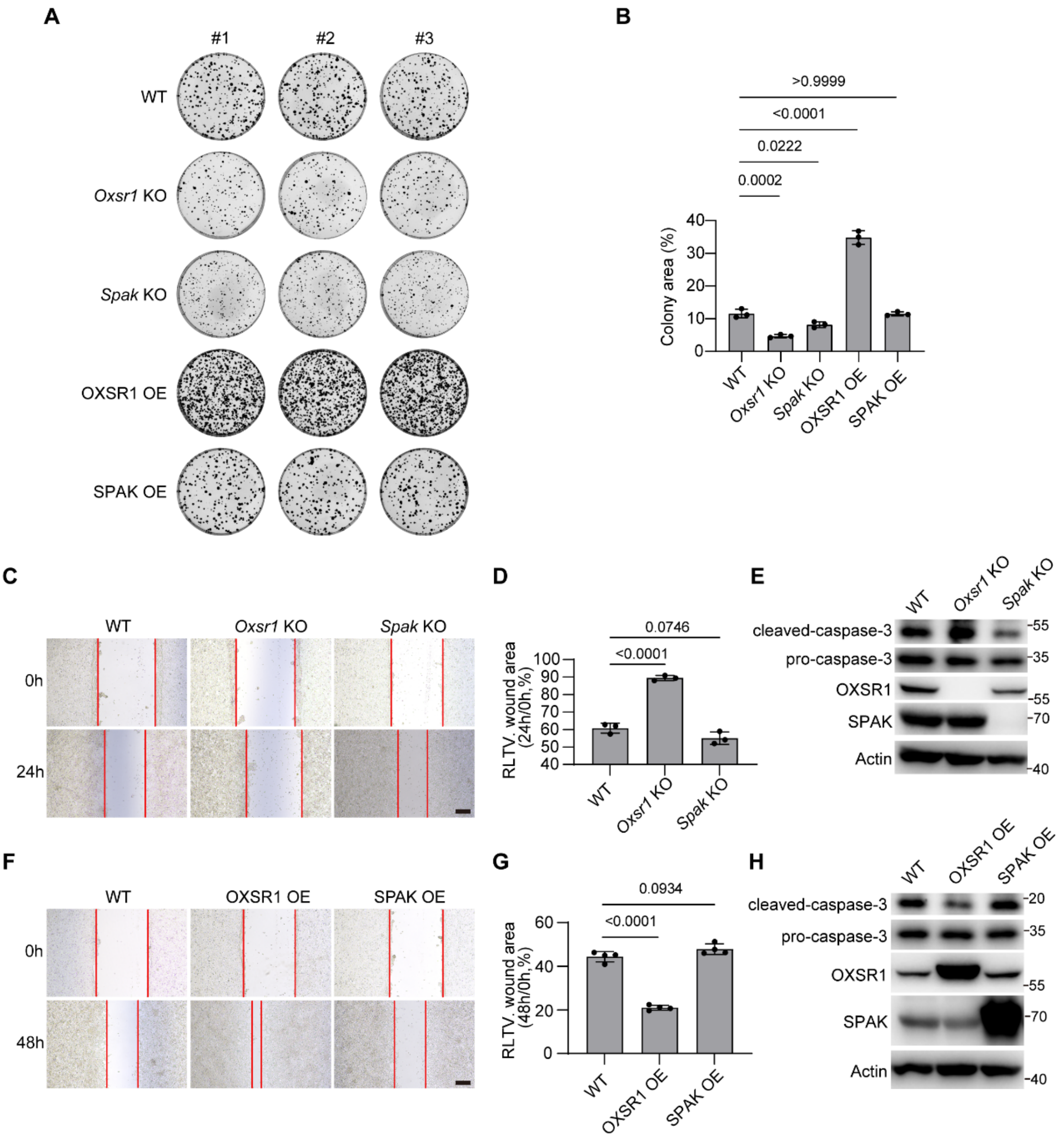

Appendix Figure S1. OXSR1 promotes cell proliferation and suppresses apoptosis in mammalian cells.

(A, B) Clonogenic assay of the HEK293T cell lines with the indicated genotypes. Representative images of colonies (A), and the quantification of colony area (B) are shown. Note that OXSR1 overexpression significantly enhanced colony areas, while *Oxsr1* knockout significantly reduced colony areas. Data were analyzed using one-way ANOVA followed by Tukey's multiple comparisons test and are presented as mean ± s.d., $n = 3$ independent experiments.

(C, D) Wound healing assay of HEK293T cells with *Oxsr1* or *Spak* knockout. Representative images of wound boundary (marked by red lines) at 0 h and 24 h (C) and the quantification of wound area (D) are shown. Note that loss of *Oxsr1*, but not *Spak*, significantly impaired wound healing. Data were analyzed using one-way ANOVA followed by Tukey's multiple comparisons test and are presented as mean ± s.d., $n = 3$ independent experiments. RLTV, relative. Scale bar: 300 μm.
(E) Western blot analysis of HEK293T cells with *Oxsr1* or *Spak* knockout. Note that loss of *Oxsr1*, but not *Spak*, significantly increased the levels of cleaved caspase-3.
(F, G) Similar to (C, D) except that HEK293T cells stably expressing OXSR1 or SPAK were used. Note that overexpression of OXSR1, but not SPAK, significantly enhanced wound healing. Data were analyzed using one-way ANOVA followed by Tukey's multiple comparisons test and are presented as mean ± s.d., $n = 4$ independent experiments. RLTV, relative. Scale bar: 300 μm.
(H) Similar to (E) except for the genotypes of the HEK293T cells. Note that overexpression of OXSR1, but not SPAK, significantly suppressed the levels of cleaved caspase-3.
Gel and microscopy images shown are representative of at least two independent experiments.

Appendix Figure S2

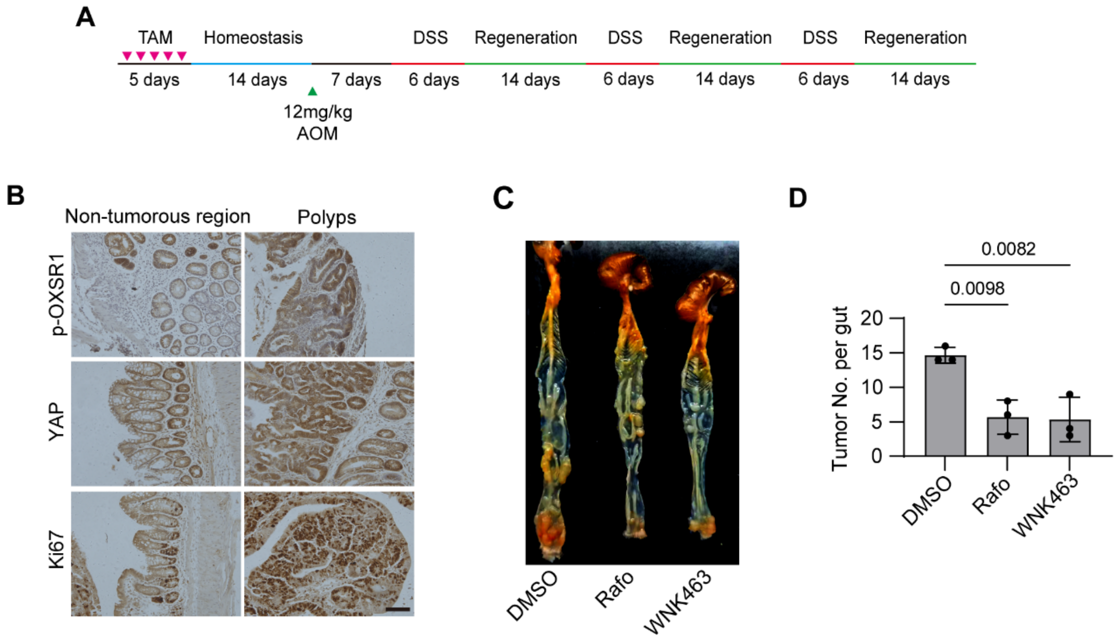

Appendix Figure S2. Targeting WNK-OXSR1 axis restricts the oncogenic potential of the intestinal regeneration program.

(A) Schematic representation of the AOM/DSS treatment regimen in mice.

(B) Immunohistochemistry was performed in polyps and the adjacent non-tumorous tissues. Note the elevated levels of OXSR1 phosphorylation, Ki67, and YAP in the polyps compared to the non-tumorous tissues. Scale bar: 100 µm.

(C, D) Representative macroscopic images of colons from mice treated with the indicated chemicals (C), and quantification of tumor numbers in the colon (D). Note that treatment with the OXSR1 inhibitor Rafoxanide (Rafo) or the WNK inhibitor WNK463 significantly reduced the tumor burden in AOM/DSS-treated mice. Data are presented as mean ± s.d. (n = 3 colons) and were analyzed using two-tailed Student's t-test.

Microscopy images shown are representative of at least two independent experiments.
