# Supplementary material for: The WNK-OXSR1 osmosensing pathway mediates intestinal regeneration via Hippo-YAP signaling
Source: EMBO J. 2026 Mar 18;45(8):2456–93. doi: 10.1038/s44318-026-00738-8 (PMC13083915; doi:10.1038/s44318-026-00738-8)
Supplement: Supplementary file 3 — Expanded View Figures [file 44318_2026_738_MOESM3_ESM.pdf]

Expanded View Figures

Figure EV1. Wnk–Fray axis regulates intestinal regeneration in *Drosophila.*

(**A**, **B**) Representative images and quantifications of adult wings (**A**) and eyes (**B**) from flies expressing Fray or *fray* RNAi under the indicated Gal4 drivers. Data were analyzed using one-way ANOVA followed by Dunnett's multiple comparisons test and are presented as mean ± s.d.; in (**A**), scale bar: 500 μm; $n = 9$ flies per group; in (**B**), scale bars: 100 μm; $n = 10$ flies per group. (**C**, **D**) Survival analysis of virgin female (**C**) and male (**D**) flies expressing a *fray* RNAi line (BDSC #42569, hereafter referred to as *fray* RNAi #2) under control of the *esg*-Gal4 driver. Flies were exposed to 5% DSS, and survival was recorded daily. Survival curves were analyzed using the log-rank (Mantel–Cox) test and are presented as mean ± s.e.m. In (**C**), $n = 32$ flies for the *esg* > GFP group and $n = 52$ flies for the *esg* > GFP; *fray*.RNAi group, pooled from three independent experiments. In (**D**), $n = 37$ flies for the *esg* > GFP group and $n = 63$ flies for the *esg* > GFP; *fray*.RNAi group, pooled from three independent experiments. (**E**) Smurf assays were performed using *fray* RNAi #2 line driven by the *esg*-Gal4 driver. Flies were treated with 3% DSS for 5 days and then fed 0.5% Brilliant Blue in 5% sucrose for 12 h before assessment. Quantification of Smurf-positive flies is shown. Data were analyzed using a two-tailed Student's *t* test and are presented as mean ± s.d.; $n = 3$ independent experiments (10 flies per experiment). (**F**) Wild-type flies were fed with DMSO, Rafoxanide (Rafo), or WNK463 and concurrently treated with 3% DSS for 2 days to induce gut injury. ISC/EB cells in the midgut were visualized using the *esg* > GFP reporter (green). Cell nuclei were counterstained with DAPI (blue). Gut boundary was marked by white dashed curves. Note that treatment with Rafo or WNK463 suppressed the DSS-induced increase in ISC/EB cell numbers. Data shown are representative images of the anterior (R2) and posterior (R4) regions of the midgut. UI uninjured. Scale bar: 50 μm. (**G**) Wild-type flies were treated as described in (**F**), but without Rafo treatment. Midguts were dissected on day 2 of the regeneration phase, stained with anti-DCP-1 antibody (red) and counterstained with DAPI (blue). White dashed curves mark the gut boundary. Scale bar: 50 μm. Data shown are representative of at least three independent experiments.

© The Author(s)

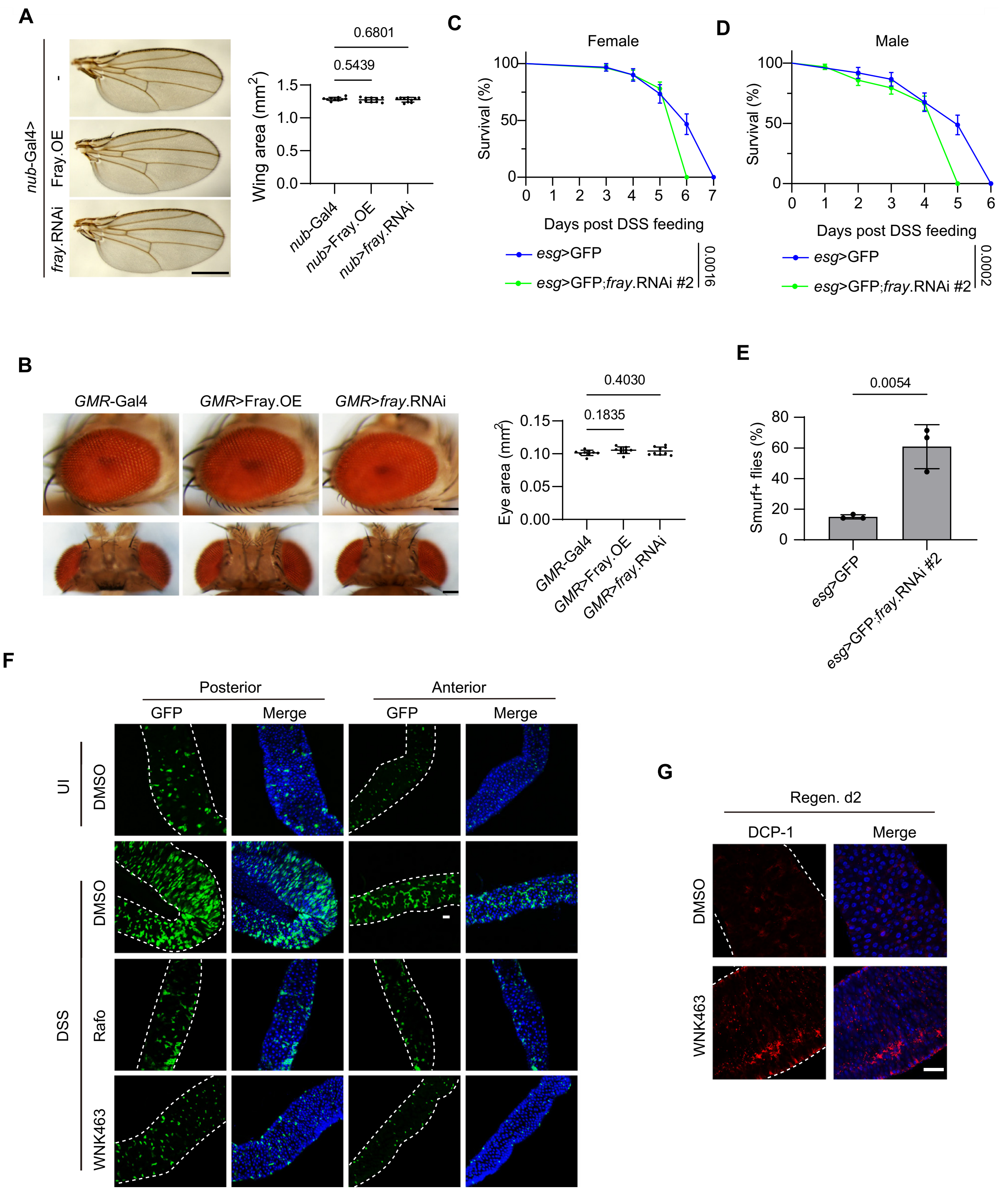
A
nub-Gal4>
-
Fray.OE
fray.RNAi
Wing area (mm2)
0.6801
0.5439
nub-Gal4
nub>Fray.OE
nub>fray.RNAi
C
Female
Survival (%)
Days post DSS feeding
esg>GFP
esg>GFP;fray.RNAi #2
0.0016
D
Male
Survival (%)
Days post DSS feeding
esg>GFP
esg>GFP;fray.RNAi #2
0.0002
B
GMR-Gal4
GMR>Fray.OE
GMR>fray.RNAi
Eye area (mm2)
0.4030
0.1835
E
Smurf+ flies (%)
0.0054
esg>GFP
esg>GFP;fray.RNAi #2
F
Posterior
Anterior
GFP
Merge
UI
DMSO
DSS
Rafo
WNK463
G
Regen. d2
DCP-1
Merge
DMSO
WNK463

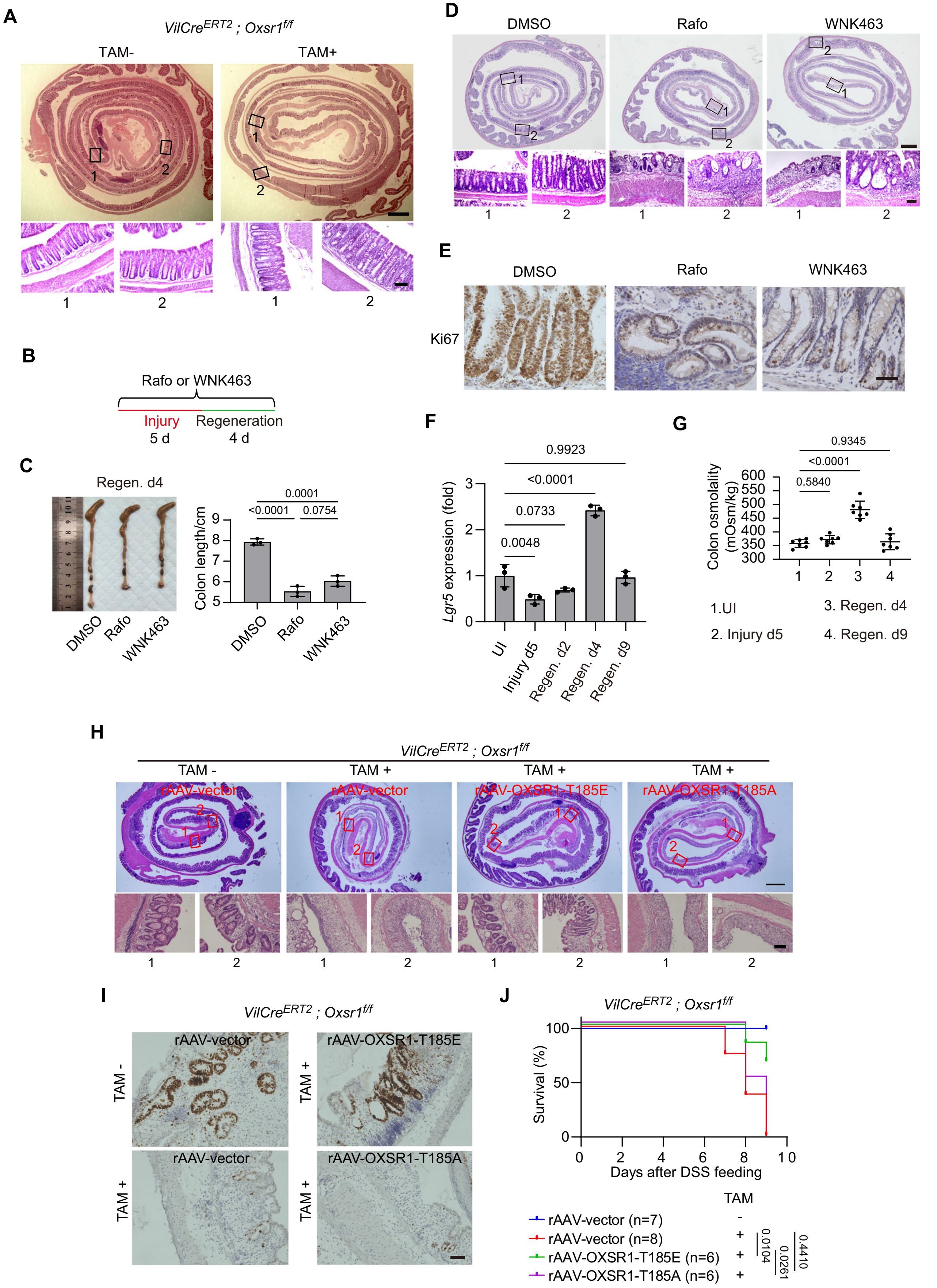
A
VilCre^ERT2 ; Oxsr1^f/f
TAM-
TAM+
B
Rafo or WNK463
Injury
5 d
Regeneration
4 d
C
Regen. d4
DMSO
Rafo
WNK463
Colon length/cm
D
DMSO
Rafo
WNK463
E
Ki67
F
Lgr5 expression (fold)
UI
Injury d5
Regen. d2
Regen. d4
Regen. d9
G
Colon osmolality (mOsm/kg)
1.UI
2. Injury d5
3. Regen. d4
4. Regen. d9
H
VilCre^ERT2 ; Oxsr1^f/f
TAM -
TAM +
rAAV-vector
rAAV-OXSR1-T185E
rAAV-OXSR1-T185A
I
J
Survival (%)
Days after DSS feeding
TAM
rAAV-vector (n=7)
rAAV-vector (n=8)
rAAV-OXSR1-T185E (n=6)
rAAV-OXSR1-T185A (n=6)

© The Author(s)

Figure EV2. WNK-OXSR1 axis regulates intestinal regeneration in mammals.

(**A**) Colon tissues were collected from mice with or without tamoxifen treatment for 5 consecutive days. Representative hematoxylin and eosin (H&E) staining images of colon Swiss rolls are shown from at least three independent experiments. Note that conditional knockout of *Oxsr1* in the intestinal epithelium did not result in any discernible abnormalities under steady-state conditions. Scale bars: 500 µm (upper panel) and 100 µm (lower panel). (**B**) Schematic representation of the chemical treatment regimen in mice. (**C**–**E**) Colons from wild-type mice with the indicated chemical treatment were collected on day 4 of the regeneration phase and subjected to similar analyses as in Fig. 2D–F. For (**C**), data were analyzed using two-tailed Student's *t* test and are presented as mean ± s.d. ($n = 3$ colons). Scale bars in (**D**): 500 µm (upper panel) and 100 µm (lower panel). Scale bar in (**E**): 50 µm. (**F**) Colonic crypts were collected at the indicated stages of injury and regeneration and *Lgr5* mRNA levels were assessed by RT-qPCR. Note that *Lgr5* mRNA levels were reduced during injury and progressively reappeared during regeneration. Data were analyzed using one-way ANOVA followed by Dunnett's multiple comparisons test and are presented as mean ± s.d. ($n = 3$ colons). (**G**) Colonic contents were collected from mice at the indicated regeneration stages. The osmolality of the colonic supernatant was measured using a Dew Point Osmometer. Note that colonic osmolality was markedly elevated during the early stage of regeneration and returned to homeostatic levels at the late stage of regeneration. Data were analyzed using one-way ANOVA followed by Tukey's multiple comparisons test and are presented as mean ± s.d., $n = 7$ mice. UI uninjured. (**H**–**J**) Recombinant adeno-associated viruses carrying the indicated OXSR1 variants were delivered to *Oxsr1* cKO mice. Colon sections were collected on day 4 of the regeneration phase and subjected to H&E (**H**) and Ki67 (**I**) staining. Mouse survival was monitored daily (**J**). Survival curves were analyzed using the log-rank (Mantel–Cox) test. *n* for each group is shown. Scale bars in (**H**): 500 µm (upper panels) and 100 µm (lower panels). Scale bar in (**I**): 50 µm.

 © The Author(s)

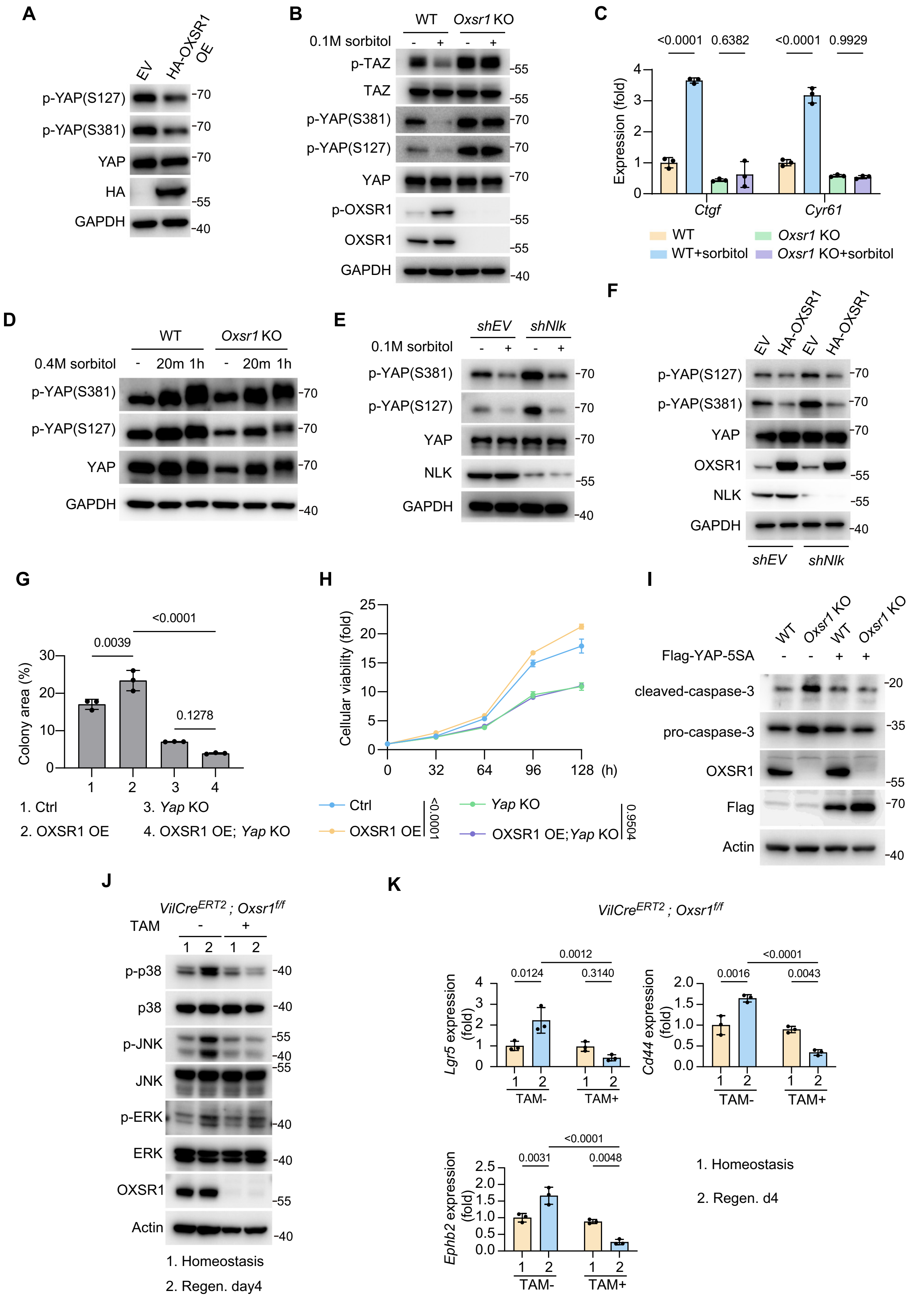
A
EV
HA-OXSR1 OE
p-YAP(S127)
p-YAP(S381)
YAP
HA
GAPDH
-70
-70
-70
-55
-40
B
WT
Oxsr1 KO
0.1M sorbitol
p-TAZ
TAZ
p-YAP(S381)
p-YAP(S127)
YAP
p-OXSR1
OXSR1
GAPDH
C
<0.0001
0.6382
<0.0001
0.9929
Expression (fold)
Ctgf
Cyr61
WT
WT+sorbitol
Oxsr1 KO
Oxsr1 KO+sorbitol
D
WT
Oxsr1 KO
0.4M sorbitol
- 20m 1h
- 20m 1h
p-YAP(S381)
p-YAP(S127)
YAP
GAPDH
E
shEV
shNlk
0.1M sorbitol
p-YAP(S381)
p-YAP(S127)
YAP
NLK
GAPDH
F
EV
HA-OXSR1
EV
HA-OXSR1
p-YAP(S127)
p-YAP(S381)
YAP
OXSR1
NLK
GAPDH
shEV
shNlk
G
<0.0001
0.0039
0.1278
Colony area (%)
1. Ctrl
2. OXSR1 OE
3. Yap KO
4. OXSR1 OE; Yap KO
H
Cellular viability (fold)
(h)
Ctrl
OXSR1 OE
<0.0001
Yap KO
OXSR1 OE;Yap KO
0.9504
I
WT
Oxsr1 KO
WT
Oxsr1 KO
Flag-YAP-5SA
cleaved-caspase-3
pro-caspase-3
OXSR1
Flag
Actin
J
VilCreERT2 ; Oxsr1f/f
TAM
p-p38
p38
p-JNK
JNK
p-ERK
ERK
OXSR1
Actin
1. Homeostasis
2. Regen. day4
K
VilCreERT2 ; Oxsr1f/f
0.0012
0.0124
0.3140
Lgr5 expression (fold)
TAM-
TAM+
<0.0001
0.0016
0.0043
Cd44 expression (fold)
<0.0001
0.0031
0.0048
Ephb2 expression (fold)
1. Homeostasis
2. Regen. d4

Figure EV3. OXSR1 regulates cell proliferation via YAP.

(**A**) Western blot was performed using HEK293T cells that stably express HA-OXSR1. Note the reduced phosphorylation of YAP upon OXSR1 overexpression. EV, empty vector. (**B**, **C**) Western blot (**B**) and RT-qPCR (**C**) were performed in HeLa cells (**B**) and HEK293T cells (**C**) with the indicated genotypes that were treated with sorbitol (0.1 M, 20 min). Note the reduced YAP/TAZ phosphorylation and enhanced YAP/TAZ target gene expression under these mild hyperosmotic stress conditions. Also note that this phenomenon was missing in *Oxsr1* knockout cells. Data in (**C**) were analyzed using two-way ANOVA followed by Tukey's multiple comparisons test and are presented as mean ± s.d., $n = 3$ independent experiments. (**D**) Western blot was performed in WT or *Oxsr1*-null HEK293T cells that were treated with sorbitol (0.4 M) for the indicated time points. Note the increased YAP phosphorylation under these intense hyperosmotic stress conditions. (**E**) Western blot analysis in HeLa cells expressing the indicated shRNAs, and were treated with sorbitol (0.1 M, 20 min). Note that knockdown of *Nlk* did not affect YAP activation induced by mild osmotic stress. (**F**) Similar to (**A**) except for the additional *Nlk* knockdown cells. Note that the reduced YAP phosphorylation caused by OXSR1 overexpression was not affected by *Nlk* depletion. (**G**) Clonogenic assay of HEK293T cell lines with the indicated genotypes. Note that the increased colony formation observed in OXSR1 overexpressing cells was completely abrogated by *Yap* knockout. Data were analyzed using two-way ANOVA followed by Tukey's multiple comparisons test and are presented as mean ± s.d., $n = 3$ independent experiments. (**H**) Cell viability of the indicated HEK293T cell lines was assessed using the CCK-8 assay. Note that the increase in viability caused by OXSR1 overexpression was completely rescued by *Yap* knockout. Data were analyzed using two-way ANOVA followed by Tukey's multiple comparisons test and are presented as mean ± s.d., $n = 3$ independent experiments. (**I**) Western blot analysis of WT or *Oxsr1* KO HEK293T cells transfected with empty vector or Flag-YAP-5SA. Note that overexpression of the constitutively active YAP-5SA abolished the increased levels of cleaved caspase-3 in *Oxsr1* KO cells. (**J**, **K**) Colon samples from *Oxsr1* cKO (TAM+) and control (TAM−) mice were collected and analyzed by western blot (**J**) or RT-qPCR (**K**). Note that the increase in the phosphorylation of p-38, JNK, and ERK, and the induction of Wnt target genes *Lgr4*, *Ephb2*, and *Cd44* in control colons during the regeneration phase were completely abolished upon *Oxsr1* knockout. For (**K**), data were analyzed using two-way ANOVA followed by Tukey's multiple comparisons test and are presented as mean ± s.d., $n = 3$ independent experiments. The gel and microscopy images shown are representative of at least two independent experiments.

 © The Author(s)

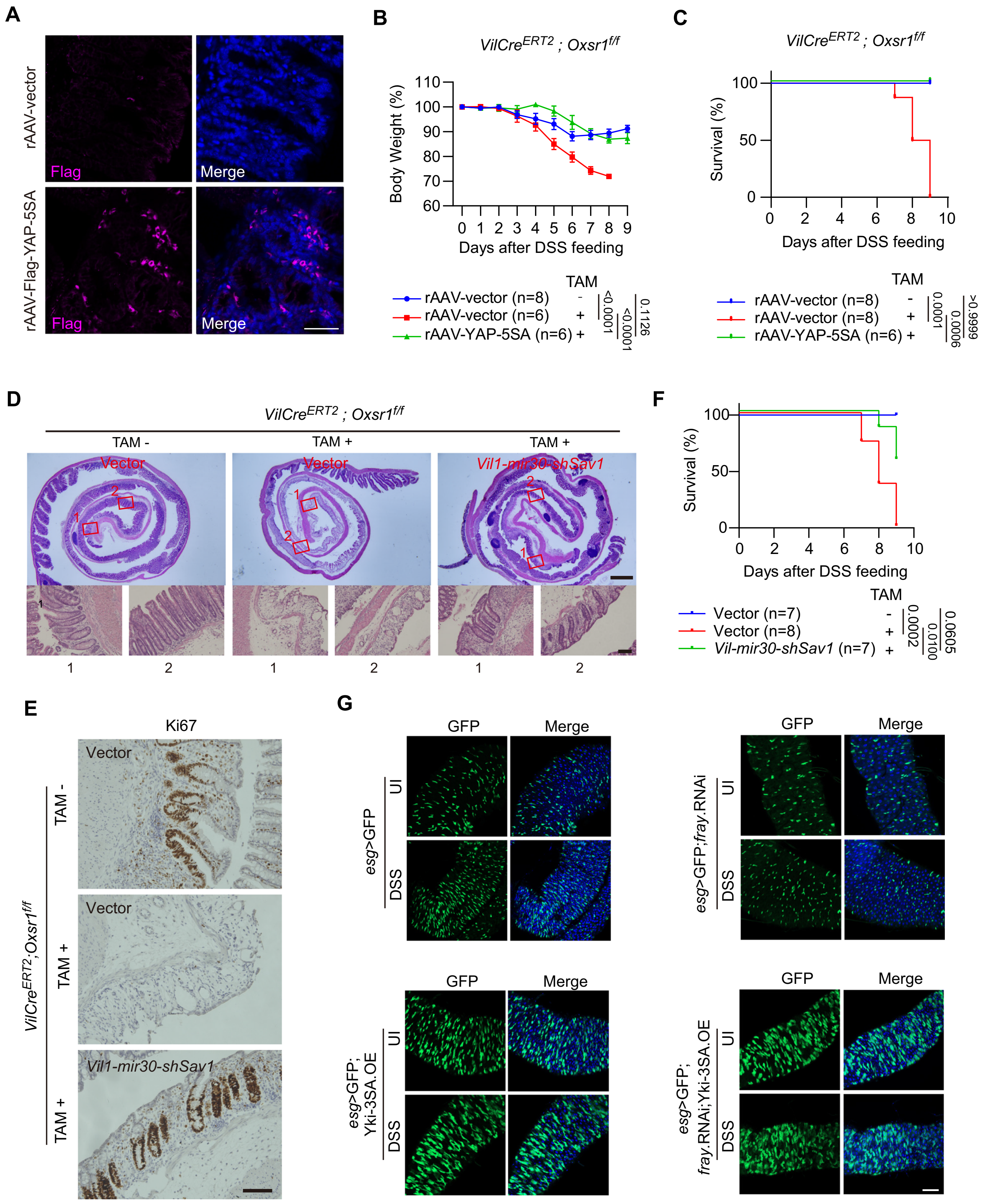
A
rAAV-vector
rAAV-Flag-YAP-5SA
Flag
Merge
B
VilCreERT2 ; Oxsr1f/f
Body Weight (%)
Days after DSS feeding
TAM
rAAV-vector (n=8) -
rAAV-vector (n=6) +
rAAV-YAP-5SA (n=6) +
<0.0001
<0.0001
0.1126
C
VilCreERT2 ; Oxsr1f/f
Survival (%)
Days after DSS feeding
TAM
rAAV-vector (n=8) -
rAAV-vector (n=8) +
rAAV-YAP-5SA (n=6) +
0.001
0.0006
>0.9999
D
VilCreERT2 ; Oxsr1f/f
TAM -
TAM +
TAM +
Vector
Vector
Vil1-mir30-shSav1
F
Survival (%)
Days after DSS feeding
TAM
Vector (n=7) -
Vector (n=8) +
Vil-mir30-shSav1 (n=7) +
0.0002
0.0100
0.0605
E
Ki67
Vector
Vector
Vil1-mir30-shSav1
TAM -
TAM +
TAM +
VilCreERT2;Oxsr1f/f
G
GFP
Merge
esg>GFP
esg>GFP;fray.RNAi
esg>GFP; Yki-3SA.OE
esg>GFP; fray.RNAi;Yki-3SA.OE
UI
DSS

Figure EV4. Activation of YAP or Yki rescues regeneration defects in *Oxsr1* cKO mice or *fray* knockdown flies.

(**A**) Delivery of rAAV-Flag-YAP-5SA to the mouse intestine was confirmed by immunostaining with anti-Flag antibody (pink). Cell nuclei were counterstained with DAPI (blue). Scale bar: 50 µm. (**B**, **C**) Body weight (**B**) and survival (**C**) of the indicated mice were monitored daily. In accordance with animal welfare guidelines, mice that lost more than 30% of their initial body weight were euthanized. Note that overexpression of YAP-5SA rescued the increased body weight loss and lethality caused by *Oxsr1* deletion. For (**B**), data were analyzed using two-way ANOVA followed by Tukey's multiple comparisons test, and are presented as mean ± s.d., *n* for each group is shown. For (**C**), survival curves were analyzed using the log-rank (Mantel–Cox) test. *n* for each group is shown. (**D**–**F**) Recombinant adeno-associated virus carrying mir30-based short-hairpin RNA targeting *Sav1* under the control of the *Vil1* promoter was delivered to *Oxsr1* cKO and control mice. Colon sections on day 4 of the regeneration phase were subjected to H&E (**D**) and Ki67 (**E**) staining. Survival of the indicated mice was monitored daily (**F**). Scale bar in (**D**): 500 µm (upper panel) and 100 µm (lower panel). Sale bar in (**E**): 50 µm. For (**F**), survival curves were analyzed using the log-rank (Mantel–Cox) test. *n* for each group is shown. (**G**) ISC/EB cells in the midguts of the indicated flies were visualized through *esg* > GFP reporter under uninjured (UI) conditions or following DSS treatment (injury day 2). Note that overexpression of a constitutively active form of Yki (Yki-3SA) restored the number of ISC/EB cells following DSS treatment in fray knockdown flies. The data shown are representative images of fly midgut (R4 region). Scale bar: 200 µm. Data shown are representative of at least two independent experiments.

 © The Author(s)

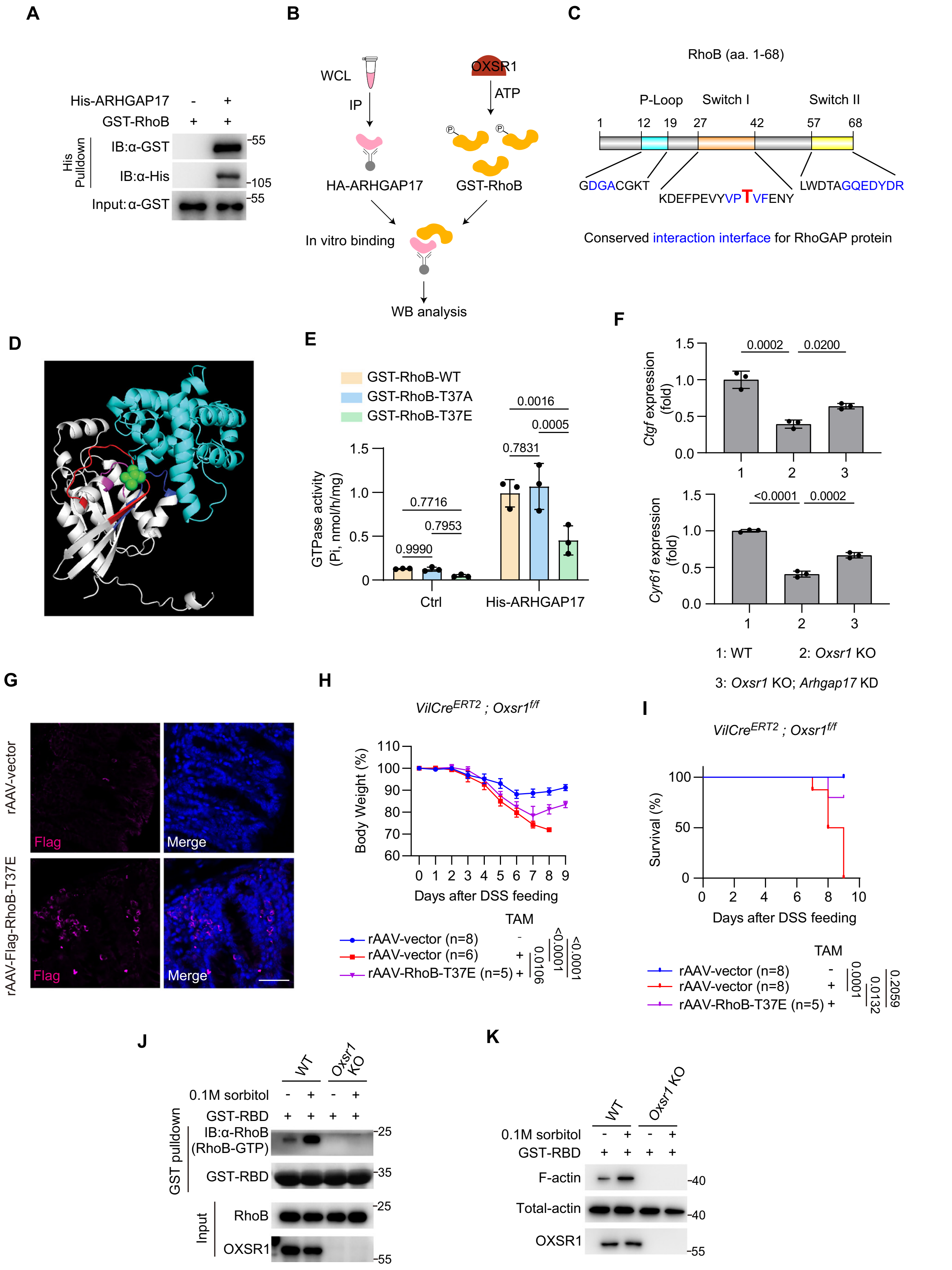
A
His-ARHGAP17 - +
GST-RhoB + +
His Pulldown
IB:α-GST
IB:α-His
Input: α-GST
B
WCL
IP
OXSR1
ATP
HA-ARHGAP17
GST-RhoB
In vitro binding
WB analysis
C
RhoB (aa. 1-68)
P-Loop Switch I Switch II
GDGACGKT
KDEFPEVYVPTVFENY
LWDTAGQEDYDR
Conserved interaction interface for RhoGAP protein
D
E
GST-RhoB-WT
GST-RhoB-T37A
GST-RhoB-T37E
GTPase activity (Pi, nmol/h/mg)
Ctrl
His-ARHGAP17
F
Ctgf expression (fold)
Cyr61 expression (fold)
1: WT 2: Oxsr1 KO
3: Oxsr1 KO; Arhgap17 KD
G
rAAV-vector
rAAV-Flag-RhoB-T37E
Flag
Merge
H
VilCreERT2 ; Oxsr1f/f
Body Weight (%)
Days after DSS feeding
TAM
rAAV-vector (n=8) -
rAAV-vector (n=6) +
rAAV-RhoB-T37E (n=5) +
I
VilCreERT2 ; Oxsr1f/f
Survival (%)
Days after DSS feeding
TAM
rAAV-vector (n=8) -
rAAV-vector (n=8) +
rAAV-RhoB-T37E (n=5) +
J
WT
Oxsr1 KO
0.1M sorbitol
GST-RBD
GST pulldown
IB:α-RhoB (RhoB-GTP)
GST-RBD
Input
RhoB
OXSR1
K
WT
Oxsr1 KO
0.1M sorbitol
GST-RBD
F-actin
Total-actin
OXSR1

Figure EV5. OXSR1 phosphorylates RhoB and inhibits its interaction with ARHGAP17.

(**A**) His pull-down assay using bacterially purified His-ARHGAP17 and GST-RhoB revealed a direct interaction between ARHGAP17 and RhoB. (**B**) Schematic illustration of the in vitro kinase assay followed by an in vitro binding assay. Bacterially purified recombinant His-OXSR1 and GST-RhoB were subjected to in vitro kinase assay, then incubated with HA-ARHGAP17 immunoprecipitated from HEK293T cell lysates. WCL, whole cell lysate. (**C**) Schematic diagram showing the conserved RhoGAP interaction interface within RhoB (aa 1–68). The T37 residue is highlighted in bold red. (**D**) Predicted 3D structure of the complex formed by human RhoB and the GAP domain of ARHGAP17 (aa 246–446), as modeled by AlphaFold. RhoB is shown in gray, with key regions highlighted: the P-loop (purple), switch I (red), switch II (blue), and residue T37 (green). The GAP domain of ARHGAP17 is depicted in cyan. (**E**) In vitro GTPase activity of GST-RhoB-WT, GST-RhoB-T37A, and GST-RhoB-T37E in the absence or presence of purified His-ARHGAP17. Purified RhoB proteins (WT, T37A, or T37E) were incubated with or without His-ARHGAP17, and GTP hydrolysis was quantified using a commercial kit (Beyotime #P2435S). Note that ARHGAP17 markedly enhanced the GTPase activity of RhoB-WT and RhoB-T37A, whereas the phosphomimetic RhoB-T37E exhibited only a modest increase. Data were analyzed using two-way ANOVA followed by Tukey's multiple comparisons test and are presented as mean ± s.d., $n = 3$ independent experiments. (**F**) RT-qPCR analysis of *Ctgf* and *Cyr61* mRNA levels in HEK293T cells of the indicated genotypes. Note that the reduction in these YAP target genes upon *Oxsr1* knockout was partially rescued by knockdown of *Arhgap17*. Data were analyzed using one-way ANOVA followed by Tukey's multiple comparisons test and are presented as mean ± s.d., $n = 3$ independent experiments. (**G**) Delivery of rAAV-Flag-RhoB-T37E to the mouse intestine was confirmed by immunostaining with anti-Flag antibody (pink). Cell nuclei were counterstained with DAPI (blue). The control group (top) is intentionally reused and corresponds to the same rAAV-vector control shown in Fig. EV4A. Scale bar: 50 µm. (**H**, **I**) Body weight (**H**) and survival (**I**) of the indicated mice were monitored daily. In accordance with animal welfare guidelines, mice that lost more than 30% of their initial body weight were euthanized. Note that overexpression of RhoB-T37E rescued the increased body weight loss and lethality caused by *Oxsr1* deletion. For (**H**), data were analyzed using two-way ANOVA followed by Tukey's multiple comparisons test and are presented as mean ± s.d., *n* for each group is shown. For (**I**), survival curves were analyzed using the log-rank (Mantel–Cox) test. *n* for each group is shown. (**J**, **K**) RhoB-GTP levels (**J**) and F-actin levels (**K**) were assessed in wild-type or *Oxsr1* knockout SW480 cells with or without sorbitol treatment (0.1 M, 1 h). Note that sorbitol treatment elevated RhoB-GTP and F-actin levels in wild-type cells, but this response was abolished in *Oxsr1*-null cells. The gel and microscopy images shown are representative of at least two independent experiments.

 © The Author(s)
